# NSAIDs for Prophylaxis for Heterotopic Ossification After Total Hip Arthroplasty: A Bayesian Network Meta-analysis

**DOI:** 10.1007/s00223-020-00763-7

**Published:** 2020-10-12

**Authors:** Filippo Migliorini, Andromahi Trivellas, Jörg Eschweiler, Arne Driessen, Markus Tingart, Nicola Maffulli

**Affiliations:** 1 Department of Orthopaedics, RWTH Aachen University Clinic, Pauwelstr. 31, 52074 Aachen, Germany; 2 Department of Orthopaedics, David Geffen School of Medicine at UCLA, 10833 Le Conte Ave, Los Angeles, CA 90095 USA; 3 Department of Medicine, Surgery and Dentistry, University of Salerno, Via S. Allende, 84081 Baronissi, SA Italy; 4 School of Pharmacy and Bioengineering, Keele University School of Medicine, Thornburrow Drive, Stoke on Trent, England; 5 Barts and the London School of Medicine and Dentistry, Mile End Hospital, Centre for Sports and Exercise Medicine, Queen Mary University of London, 275 Bancroft Road, London, E1 4DG England

**Keywords:** Heterotopic ossification, Total hip arthroplasty, NSAID

## Abstract

Non-steroidal anti-inflammatory drugs (NSAID) have been recommended to prevent of heterotopic ossification (HO) after total hip arthroplasty (THA), but debates are still ongoing. The present Bayesian network meta-analysis of randomized clinical trials (RCTs) compared all available pathways of NSAID treatment as prophylaxis for HO after THA. The present Bayesian network meta-analysis was conducted according to The PRISMA Extension Statement for Reporting of Systematic Reviews Incorporating Network Meta-analyses of Health Care Interventions guidelines. All randomized clinical trials comparing two or more interventions to prevent HO after THA were considered for analysis. HO was classified according to Brooker. The quality of the methodological assessment was performed through the risk of bias summary tool of the Review Manager Software 5.3 (The Cochrane Collaboration, Copenhagen). The network meta-analysis was performed through a STATA routine for Bayesian hierarchical random-effects model analysis, with log odd ratio (LOR) effect measure. Data from 26 studies (6396 THAs; 58% females) were collected. The mean follow-up was 10.50 ± 5.7 months. ANOVA showed good comparability among mean age and gender (*P* > 0.5). Celecoxib demonstrated the highest rate of Brooker class 0 (LOR 6.96), followed by diclofenac (LOR 6.94). Naproxen demonstrated the lowest rate of Brooker I HO (LOR 2.82), followed by celecoxib (LOR 3.52). Celecoxib demonstrated lowest rate of Brooker class II HO (LOR 1.66), class III (LOR), and class IV (LOR 0.25). The equation for global linearity detected no statistically significant inconsistency (*P* > 0.5) in all the comparisons. The present Bayesian network meta-analysis encourages the use of celecoxib as a prophylaxis for HO. These conclusions must be interpreted in light of the limitations of the present study. Future investigations are required to establish more definitely the role of celecoxib.

Level of Evidence: I, Bayesian network analysis of RCTs.

## Introduction

Heterotopic ossification (HO) is common after total hip arthroplasty (THA) [1], and is characterized by the formation of ectopic bone within the surrounding muscle and soft tissues. If no prophylactic treatment is implemented, the incidence of HO ranges between 15 and 90% [2–4]. In patients with high risk of HO, radiotherapy is recommended [5]. Alternatively, NSAID have been recommended to prevent HO after THA [6]. Several studies compared the use of NSAIDs against HO after THA [7–11], but the most effective prophylactic treatment remains elusive, and no evidence-based guidelines to prevent HO after THA are available. Several meta-analyses have been performed, but the drug of choice has not yet been identified, and debates are ongoing. The limit of these articles is intrinsic in the statistical nature of meta-analyses, which allows to compare only two treatments for the same intervention. Differently, in network meta-analyses multiple treatments ($\ge$3) can be compared using both direct comparisons of interventions within RCTs and indirect comparisons across trials [12]. Therefore, we performed a Bayesian network meta-analysis of RCTs comparing all the available NSAID treatments as prophylaxis for HO after THA to identify the most suitable drug(s) for prophylaxis. This study adds to the existent literature evidenced-based recommendations concerning the optimal pharmacological strategy to prevent HO after THA.

## Materials and Methods

### Search Strategy

The present Bayesian network meta-analysis was conducted according to the PRISMA Extension Statement for Reporting of Systematic Reviews Incorporating Network Meta-analyses of Health Care Interventions guidelines [13]. A primary protocol was established:

- P (patients): total hip arthroplasty;
- I (intervention): prevention of HO;
- C (comparison): oral therapy drugs;
- O (outcomes): grade of HO;
- S (study type): randomized clinical trial (RCT).

### Literature Search and Data Extraction

Two independent authors (AD, FM) performed the literature search in September 2020. First, the following databases were accessed: Pubmed, Embase, Scopus, Google Scholar. The search covered from initiation of the database to September 2020. The following keywords were used in combination: total hip arthroplasty, replacement, prosthesis, heterotopic ossification, NSAID, COX-inhibitors, impingement, indomethacin, naproxen, acetylsalicylic acid, celecoxib, meloxicam, rofecoxib, ibuprofen, diclofenac. Two independent authors (AD, FM) performed data extraction. If the title and abstract matched the topic, the full-text was accessed. The bibliographies of the considered articles were also screened for inclusion. Disagreements were debated and solved by a third author (MT).

### Eligibility Criteria

All randomized clinical trials comparing two or more *interventions used* to prevent HO formation were considered for analysis in the present study. According to the authors’ language capabilities, articles in English, French, German, Italian, Portuguese and Spanish were considered. Only level I of evidence RCTs according to the Oxford Centre of Evidenced-Based Medicine [14] were included. Editorials, cohort studies, review and meta-analyses, expert opinion and letters were excluded. Animals, biomechanics, cadaveric and in vitro studies were also excluded. Grades of HO were evaluated using the Brooker classification [15]. Other classification systems were not considered in the present study. Protocols for prevention of HO using ionizing radiations were not considered in the present study. Only articles reporting quantitative data concerning the outcomes of oral drug consumption to prevent HO were included in the present study. Missing data under the outcomes of interest warranted the exclusion from the present network meta-analysis.

### Data extraction and Outcomes of Interest

Data extraction was performed by two independent authors (AD, FM). The following data were collected: generalities of the studies (author, year), duration of the follow-up (months), type of treatment and related protocol, number of samples, mean age and percentage of females among the study cohort. The outcome of interest was to evaluate the effect of oral non-steroidal anti-inflammatory drugs to prevent HO defined according to the modified Brooker Staging System (Table 1). This classification differs from the original by an additional grade 0, in which there is no sign of HO [16].

**Table 1:** Modified Brooker Staging System

Class	Radiographic findings
Grade 0	No sign of heterotopic ossification
Grade I	Bone islands in the soft tissue around the hip
Grade II	Exophytes in the pelvis or proximal end of the femur with at least 1 cm between opposing bone surfaces
Grade III	Exophytes in the pelvis or proximal end of the femur with less than 1 cm between opposing bone surfaces
Grade IV	Bony ankylosis between proximal femur and pelvis

### Methodological Quality Assessment

The quality of the methodological assessment was performed through the risk of bias summary tool of the Review Manager Software 5.3 (The Cochrane Collaboration, Copenhagen). For the present analysis, six items from each study were evaluated: allocation, randomization, blinding of the assessors, selective reporting, incomplete data, and unknown source of bias.

### Statistical Analysis

The statistical analysis was performed by the first author (FM). For baseline comparability, the ANOVA test was performed using the IBM SPSS Software version 25, with a *P* > 0.5 considered statistically significant. Analytical statistics was performed using the STATA Software/MP, Version 16 (Stata Corporation, College Station, Texas, USA). The same software was used to produce an additional graphic (Fig. 4) that displays the results, specifically of the rate of HO according to the modified Brooker classification for each drug. The network meta-analyses were performed through a Stata routine for Bayesian hierarchical random-effects model analysis. For the binary data, the effect estimates were evaluated through the natural logarithm of the odds ratio (LOR) statistical method [17]. Placebo was not considered as proper reference. Rather, the comparisons were matched to a reference group of “no event”. Thereby, the final effect of each treatment ranks with respect to the reference group “no event”. The overall inconsistency was obtained through the equation for global linearity via the Wald test. If the *P* value > 0.5, the null hypothesis cannot be rejected, and the consistency assumption could be accepted at the overall level of each treatment. Both confidence (CI) and percentile (PrI) intervals were set at 95%. Edge plots were performed in all comparisons to evaluate the amount of direct and indirect observations. Interval plots were produced in all comparisons to rank the treatments according to the reference value and related effect size. Funnel plots were generated to estimate the risk of publication bias by plotting the natural logarithm of an individual study effect size against the standard error of the natural logarithm of an individual study effect size. This methodology has been already used in previous studies [18–21]. A further meta-analysis comparing subgroups selective vs non-selective NSAID was performed. The STATA Software/MP was used. For the comparison, the Mantel–Haenszel statistic method for dichotomous data was adopted, with odds ratio (OR) effect measure. The CI was set at 95%. The *Higgins-I*^2^ test was performed to evaluate heterogeneity. If the *Higgins-I*^2^ test was > 50%, high heterogeneity was detected, the data were analysed through a random model effect. If the *Higgins-I*^2^ test was < 50%, a fixed effect model was adopted. *P* values < 0.05 were considered statistically significant.

## Results

### Search Result

**Fig. 1:**
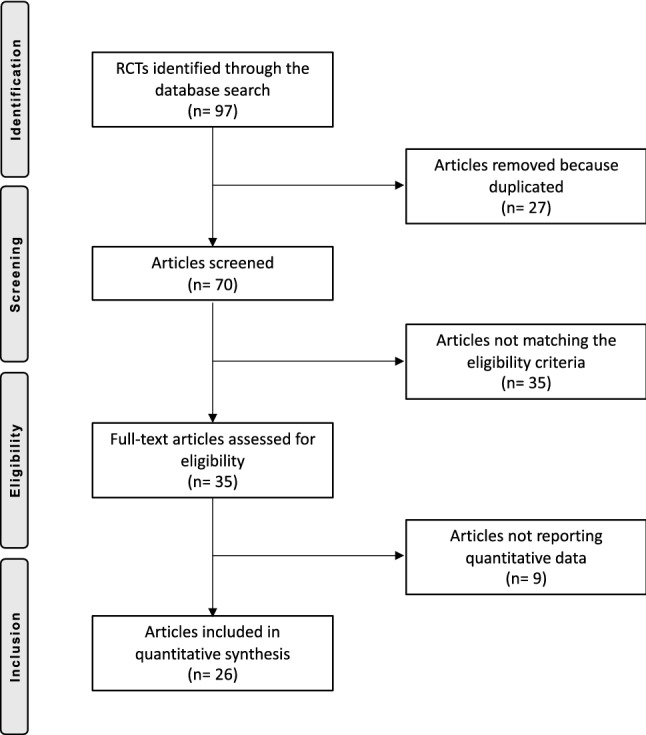
PRISMA Literature search flow chat

The literature search resulted in 302 articles, of which 97 were RCTs. After removal of duplicates (*N* = 27), a further 32 articles were excluded either because they did not report quantitative data (*N* = 9), language limitation (*N* = 3), animal or biomechanical or cadaveric studies (*N* = 12), focused only on radiation (*N* = 18), or delivered uncertain data (*N* = 2). Ultimately, 26 RCTs were included in the present study. The flow chart of the literature search is shown in Fig. 1.

### Methodological Quality Assessment

**Fig. 2:**
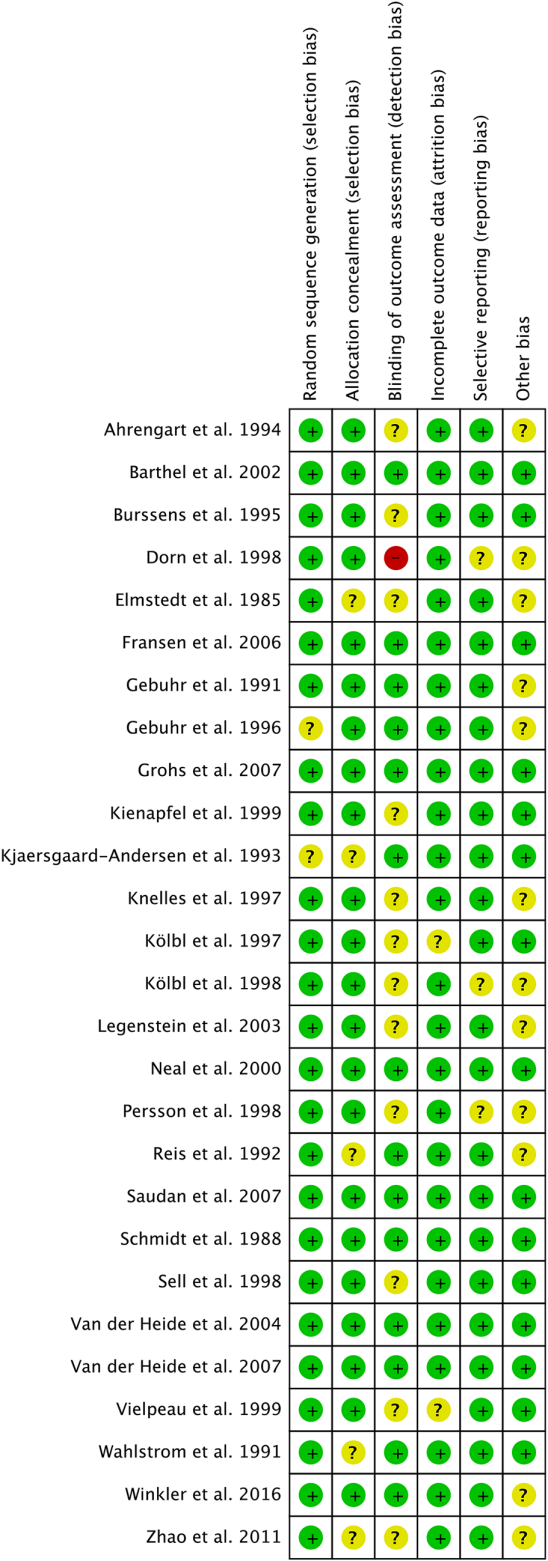
Cochrane risk of bias summary tool

In concert with the above-mentioned assessment of risk of bias, a low risk of selection bias can be evidenced. Similarly, attrition and reporting bias can be considered a moderate to low risk. The risk of unknown bias is also moderate to low. Therefore, the methodological assessment of this work can be judged as a very good quality. The Cochrane risk of bias summary tool is shown in Fig. 2.

### Risk of Publication Bias

**Fig. 3:**
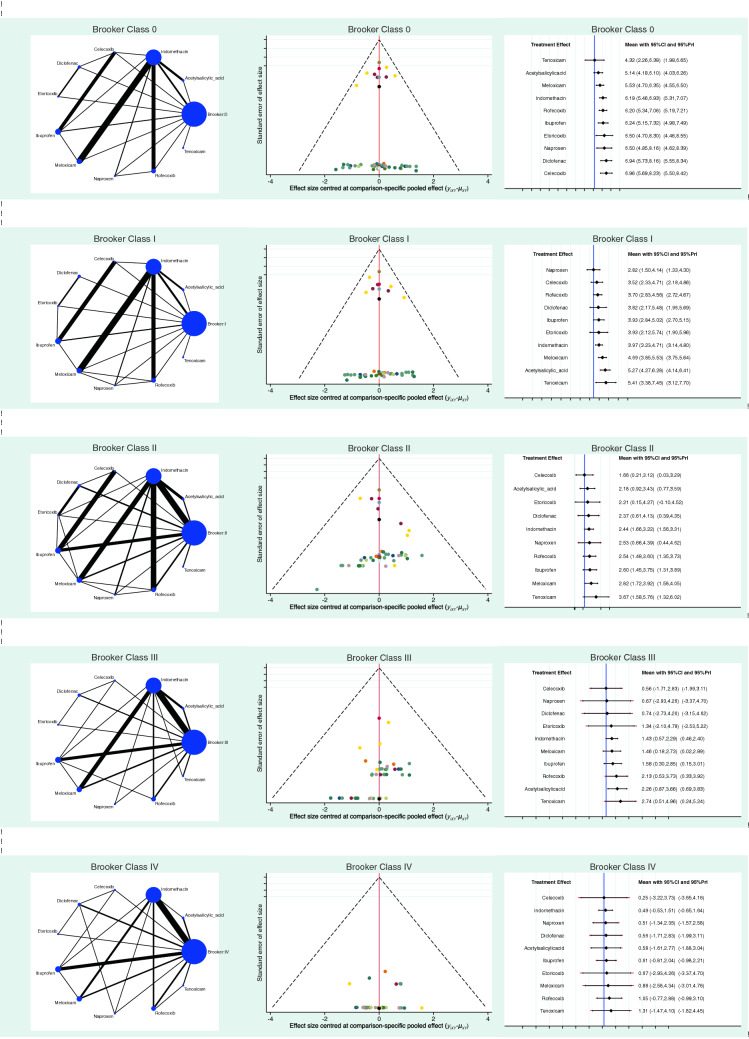
Overall results: edge, interval, and funnel plots of the network comparisons. Celecoxib, diclofenac and naproxen are the drugs with the higher rate of “no sign of HO” (modified Brooker class 0), and also those with the lowest rate of HO signs in the Brooker classes I, II, III, IV

The analysis of the funnel plots detected good symmetrical distribution of the referral points. All referral points among the funnel plots were under the range of acceptability. The risk of publication bias was low. The funnel plots are reported in Fig. 3.

### Patient Demographics

Data from 6396 (58% females) THAs were collected. The mean follow-up was 10.50 ± 5.7 months. The mean age of the patients was 66.30 ± 3.6 years. The mean duration of the drug administration for HO prevention was 20.95 ± 18.3 days. ANOVA showed good comparability in mean age and gender (*P* > 0.5). Table 2 shows the demographic baseline of the studies, while Table 3 shows the daily dose and treatment duration of each drug.

**Table 2:** Demographic baseline of the studies (NR: not reported)

Author, year	Follow-up (*months*)	Type of treatment	Type of protocol	Samples (*n*)	Mean age	Female gender (%)
Ahrengart et al. 1994 [22]	12	Ibuprofen	1500 mg daily/9 days	21	70.0	52.6
		Placebo	Placebo	26	70.0	52.6
Barthel et al. 2002 [23]	12	Meloxicam	7.5 mg daily/14 days	24	65.0	42.3
		Meloxicam	15mg daily/14 days	115	63.0	65.0
		Indomethacin	100 mg daily/14 days	111	63.0	64.2
Burssens et al. 1995 [24]	6	Tenoxicam	10 mg daily/42 days	27	61.0	
		Tenoxicam	20 mg daily/42 days	26	59.0	
		Placebo	Placebo	27	62.0	
Dorn et al. 1998 [25]	12	Indomethacin	150 mg daily/4 days	104		61.5
		Indomethacin	150 mg daily/8 days	105		60.0
Elmstedt et al. 1985 [26]	12	Ibuprofen	1200 mg daily/92 days	21	70.0	52.4
		Placebo	Placebo	21	70.0	60.0
Fransen et al. 2006 [27]	12	Ibuprofen	1200 mg daily/14 days	391	66.0	45.0
		Placebo	Placebo	407	67.0	45.6
Gebuhr et al. 1991 [28]	12	Naproxen	750 mg daily/28 days	28	75.0	60.7
		Placebo	Placebo	27	70.0	55.5
Gebuhr et al. 1996 [29]	12	Tenoxicam	40/20 mg daily/5 days	61	72.0	
		Placebo	Placebo	62	72.0	
Grohs et al. 2007 [30]	12.0	Rofecoxib	25 mg daily/7 days	50	60.0	66.0
		Indomethacin	100 mg per daily/7 days	50	60.0	60.0
Kienapfel et al. 1999 [31]	18	Indomethacin	100 mg daily/42 days	55	64.4	60.0
		Control group	No treatment	50	66.0	76.0
Kjaersgaard-Andersen et al. 1993 [32]	3	Indomethacin	100 mg daily/14 days	34	72	68.4
		Placebo	Placebo	34	70	63.6
Knelles et al. 1997 [33]	12	Indomethacin	100 mg daily/14 days	90	67.0	68.0
		Indomethacin	100 mg daily/7 days	113	64.7	72.0
		Acetylsalicylic acid	2250 mg daily/14 days	93	66.5	
		Control group	No treatment	100	65.3	69.0
Kölbl et al. 1997 [34]	12	Indomethacin	100 mg daily/7 days	113	64.7	63.7
		Control group	No treatment	100	65.3	69.0
Kölbl et al. 1998 [35]	6	Diclofenac	150 mg daily/14 days	54	63.9	51.8
		Control group	No treatment	100	65.3	69.0
Legenstein et al. 2003 [36]	6	Indomethacin	150 mg daily/12 days	58	68.0	59.0
		Meloxicam	7.5 mg daily/12 days	58	65.0	74.0
Neal et al. 2000 [37]	9	Acetylsalicylic acid	162 mg daily/35 days	1039	66.0	50.0
		Placebo	Placebo	1009	65.0	51.0
Persson et al. 1998 [38]	12	Ibuprofen	1200 mg daily/21 days	48		50.0
		Ibuprofen	1200 mg daily/7 days	48		50.0
		Placebo	Placebo	47		53.3
Reis et al. 1992 [39]	24	Diclofenac	150 mg daily/42 days	80		
		Placebo	Placebo	80		
Saudan et al. 2007 [40]	3	Celecoxib	400 mg daily/10 days	117	69.0	53.0
		Ibuprofen	1200 mg daily/10 days	123	70.0	54.0
Schmidt et al. 1988 [41]		Indomethacin	75 mg daily/42 days	102	67.0	
		Placebo	Placebo	99	68.0	
Van der Heide et al. 2004 [42]	6	Indomethacin	150 mg daily/7 days	89	67.0	68.5
		Meloxicam	15 mg daily/7 days	92	67.0	68.5
		Control group	No treatment	170		
Van der Heide et al. 2007 [43]	12	Indomethacin	150 mg daily/7 days	89		62.4
		Rofecoxib	50 mg daily/7 days	85		62.4
Vielpeau et al. 1999 [44]	6	Naproxen	750 mg daily/42 days	28	66.0	
		Indomethacin	75 mg daily/42 days	28	63.9	
		Placebo	Placebo	28	62.8	
Wahlstrom et al. 1991 [45]	24	Diclofenac	150 mg daily/42 days	50	71.0	40.0
		Placebo	Placebo	50	70.0	39.1
Winkler et al. 2016 [46]	6	Diclofenac	150 mg daily/9 days	44	61.9	45.8
		Etoricoxib	90 mg daily/9 days	45	60.2	46.8
Zhao et al. 2011 [47]	1.5	Celecoxib	200 mg daily/42 days	25	65.4	
		Indomethacin	75 mg daily/42 days	25	65.4

**Table 3:** Dose and duration of the therapy

Drug	Daily administration (mg)	Mean duration of assumption (days)
Acetylsalicylic acid	162	35.0
Acetylsalicylic acid	2250	14.0
Celecoxib	200	42.0
Celecoxib	400	10.0
Diclofenac	150	26.75
Etoricoxib	90	9.0
Ibuprofen	1500	9.0
Ibuprofen	1200	28.8
Indomethacin	75	42.0
Indomethacin	100	15.0
Indomethacin	150	7.6.0
Meloxicam	7.5	13.0
Meloxicam	15	10.5
Naproxen	750	35.0
Rofecoxib	25	7.0
Rofecoxib	50	7.0
Tenoxicam	10	42.0
Tenoxicam	20	42.0
Tenoxicam	30	5.0

### Outcomes of Interest

**Fig. 4:**
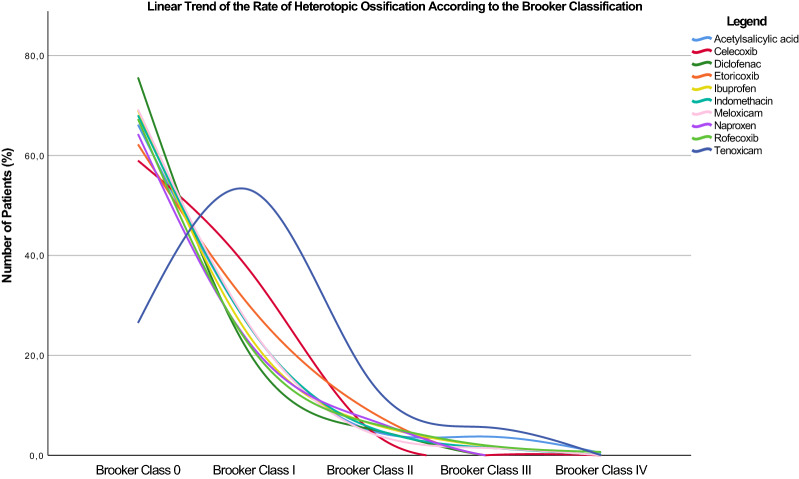
Rate of HO according to the modified Brooker classification for each drug. Celecoxib followed by naproxen and diclofenac are those that have the greatest tendency to reduce according to the progression of the Brooker classes

Celecoxib demonstrated the highest rate of modified Brooker class 0 (LOR 6.96; 95% CI 5.69 to 8.23), followed by diclofenac (LOR 6.94; 95% CI 5.73 to 8.16). Naproxen demonstrated the lowest rate of HO according to the Brooker class II (LOR 2.82; 95% CI 1.50 to 4.14), followed by celecoxib (LOR 3.52; 95% CI 2.33 to 4.71). Celecoxib demonstrated the lowest rate of HO according to the Brooker class II (LOR 1.66; 95% CI 0.21 to 3.12), followed by acetylsalicylic acid (LOR 2.18; 95% CI 0.92 to 3.43). Celecoxib demonstrated the lowest rate of HO according to the Brooker class III (LOR 0.56; 95% CI − 1.71 to 2.83), followed by naproxen (LOR 0.67; 95% CI − 2.93 to 4.26). Celecoxib demonstrated the lowest rate of HO according to the Brooker class IV (LOR 0.25; 95% CI − 3.22 to 3.73), followed by indomethacin (LOR 0.49; 95% CI − 0.53 to 1.51). The equation for global linearity detected no statistically significant inconsistency (*P* > 0.5) in all the comparisons. These results are shown in greater detail in Fig. 3, while Fig. 4 displays the rate of the HO according to the modified Brooker classification for each drug.

### Subgroup Analysis: Non-selective NSAIDs Versus Selective NSAIDs

Selective NSAIDs compared to the non-selective NSAIDs resulted not significant in all the comparisons: Brooker class 0 (OR 1.68; 95% CI 0.97 to 2.90; *P* = 0.6), class I (OR 0.74; 95% CI 0.46 to 1.20; *P* = 0.2), class II (0.82; 95% CI 0.60 to 1.10; *P* = 0.2), class III (OR 1.07; 95% CI 0.66 to 1.73; *P* = 0.8), and class IV (OR 2.06; 95% CI 0.46 to 9.16; *P* = 0.3).

## Discussion

The present Bayesian network meta-analysis demonstrated that prophylaxis with celecoxib was associated with the lowest rate of HO after THA, followed by diclofenac and naproxen. On the other hand, tenoxicam, acetylsalicylic acid, and meloxicam were associated with the highest rate of HO following THA. Subgroup analysis of COX-2 selective versus non-selective NSAID demonstrated no statistically consistent difference.

A recent network meta-analysis included also radiation, which resulted to be the most effective method to prevent HO [48]. However, radiation is recommended only for patients at high risk: bilateral hypertrophic osteoarthritis, prior history of HO, and arthritis caused by trauma characterized by hypertrophic osteophytosis [5, 49]. Potential adverse effects of radiation involve wound healing delays, fatigue and joint swelling. Trochanter non-union has been observed in 12% to 30% of patients after radiation [50]. Radiation may prevent acetabulum or proximal femur bone ingrowth, leading to failure in cementless and porous implants [5]. In male patients, even with low doses and testicular shielding, there is concern that radiation can reduces sperm count and activity [5]. Even if rare, secondary malignancies developed after hip irradiation [51, 52]. Regardless of the prophylaxis for HO, for selected patients following THA post-operative NSAID pain therapy is often administered. Thus, prevention of HO via NSAID offers a comfortable and safe alternative.

In the present network meta-analysis, celecoxib showed powerful capability to reduce HO. Neal et al. [40], analysing celecoxib in a randomized study of 240 patients, found that a post-operative ten-day prophylaxis regimen reduced the risk of Brooker grade I HO by 50%, and grades II and III by 75% compared to ibuprofen. In 2014, Lavernia et al. [53] analysed over 154 patients retrospectively, and found statistically significant lower rates of HO in the celecoxib cohort compared to the control group.

Similar results were found in a case–control study by Oni et al. [54]. In the present study, diclofenac yielded powerful capability to prevent HO. Two RCTs [35, 55], including in total 354 patients showed that diclofenac was as effective at preventing HO compared to radiation. In 2016, Winkler et al. [46] performed a prospective, double-blinded RCT comparing diclofenac versus etoricoxib: the two drugs were equally effective. Most other studies analysed indomethacin and ibuprofen. Among the various studies included, no consensus was demonstrated. Thus, it was no possible to analyse related protocols separately. This may increase the risk of bias and heterogeneity. Indeed, even in our study the heterogeneity was high, resulting in a wide CI of the outcomes.

Both ibuprofen and indomethacin are moderately capable to prevent HO. Two double-blinded RCTs [22, 27], collecting in total data from 949 patients, found no effect of ibuprofen on HO. Conversely, a double-blind RCT [42] detected reduced HO in patients treated with ibuprofen, but no dose- or time-correlation was found. An RCT comparing 240 patients receiving celecoxib versus ibuprofen evidenced a reduced rate of HO development in the celecoxib cohort [40]. Another RCT (209 patients) [25] reported less severe HO in the indomethacin cohort compared to the control group. Van der Heide et al. [42, 43] reported comparable results with indomethacin versus rofecoxib and versus meloxicam.

In the present study, the use of naproxen as prophylaxis for HO was controversial. Although optimal in terms of Brooker 0 and I classes, naproxen is also correlated with a high increase of Brooker class III and IV HO, showing heterogeneous values and wide CI. Therefore, these data are not reliable and must be interpreted with caution. Vielpeau et al. [44] observed the efficacy of naproxen in a cohort of 84 patients, and found that naproxen is an effective and safe prophylaxis for HO, and results were comparable to those observed with indomethacin. Further studies are required to investigate the role of naproxen as prophylaxis for HO.

In the present network meta-analysis, the effect of rofecoxib was moderate. Comparing Brooker class I HO, rofecoxib was comparable to celecoxib and diclofenac. However, data on this drug have been reported with high variability. Two RCTs enrolling 286 patients analysing the effect of rofecoxib found no differences when it was compared to indomethacin [30, 43]. Similarly, fair results were reported with etoricoxib. Etoricoxib evidenced medium capability in the comparison of Brooker class 0 and I, but good capability in the comparison of Brooker class II and III. Concerning Brooker class IV HO, etoricoxib produced heterogeneous results and scored moderately. However, given the heterogeneous results of etoricoxib and rofecoxib, data from these comparisons should be interpreted with caution.

The present network meta-analysis showed that acetylsalicylic acid produced heterogeneous results, and provided moderate to fair capability of it to inhibit HO compared to other NSAIDs. Two RCTs including 2733 patients tested the efficacy of acetylsalicylic acid [33, 37]. The study *interventions* were different, but both investigations agreed that it had no major effect on heterotopic bone formation, and the balance of risks and benefits does not justify the use of acetylsalicylic acid for this purpose. Assessing meloxicam and tenoxicam, a fair efficacy to prevent HO was detected. Tenoxicam efficaciously prevented HO in two different drug administration protocols in a double-blind placebo RCT [24]. However, in our network comparisons, tenoxicam was not superior to the other drugs. Several authors evaluated the efficacy of meloxicam. A RCT comparing meloxicam versus indomethacin in a cohort of 272 patients found a statistically significant higher rate of HO in the meloxicam cohort [23]. Legenstein et al. [36] found no significant difference between meloxicam versus indomethacin in 116 patients. Similar results were found by Van der Heide et al. [42] in 182 patients. Accordingly, the evidence in favour of the use of tenoxicam and meloxicam for HO prevention is dubious.

Results from the subgroup analysis are in agreement with recent meta-analyses. In 2018, Zhu et al. [56] performed a meta-analysis comparing COX-2 selective NSAIDs versus non-selective NSAIDs. Analysing data from 1636 patients (8 RCTs), no differences were found between the two classes of medications. Similar results were found by Joice et al. [57] in 2018 analysing data from 29 studies (6695 patients). Similarly, Kan et al. [1] did not find differences among the two groups of drugs in 5995 patients. Furthermore, Grohs et al. [30] analysed the Harris hip score among patients treated with non-selective and selective COX-2 inhibitor NSAIDs for HO, evidencing no statistically significant differences. Given these comparable results, and the use of selective NSAIDs being associated with less side effects and post-operative bleeding compared to non-selective NSAIDs, their use should be encouraged [56–58]. *To avoid untoward events, selective NSAIDs must be administered with caution in patients with cardiovascular risk.*

The present network meta-analysis was precise and detailed, but this study has limitations. Firstly, the drug administration protocols analysed were different from one study included to the other. The high variability in treatment protocols produced high heterogeneity in the included articles. Therefore, conclusion from the present work must be interpreted with caution. Another important limitation is the relatively small number of RCTs eligible for inclusion, reflecting the lack of evidence in the published literature on this topic. Further high-quality studies are required to definitively establish the role of non-selective NSAIDs and administration protocols. Because of the lack of quantitative data, several drugs (e.g. calcitonin, flurbiprofen, ketorolac, ketoprofen) were not considered in the present study, representing another limitation. All the included articles, even those with follow-up shorter than 12 months, referred to the Brooker classification. We also must underline that, although HO formation generally is detectable early after surgery, its extent and Brooker grade can not be definitively assessed until 12 months after surgery. All the drugs were compared regardless to their daily intake or therapy duration. This represents an important limitation of the present study. As shown in Table 3, the daily intake and therapy duration were highly variable, increasing the risk of selection bias. This reflected the limited available data in the literature. We must further acknowledge that many studies are older than 20 years, and only two have been published in the last decade (2010–2019). In the light of these limitations, given the aforementioned controversies and the lack of consensus, the results of the present Bayesian network meta-analysis must be interpreted with caution. Future studies should progress from our results and conduct high-quality trials investigating, for example, the potential costs and benefits of Celecoxib as prophylaxis for HO.

## Conclusion

In total hip arthroplasty, the use of celecoxib as prophylaxis was associated with the lowest rate of HO. Tenoxicam, acetylsalicylic acid, and meloxicam were associated with the highest rates of HO. Subgroup analysis of COX-2 non-selective versus selective COX-2 inhibitors NSAIDs showed no statistically consistent difference. These conclusions must be interpreted within the limitations of the present study. Further investigations are required to improve current evidences.
